# Influence of Dorper lamb development from birth to 120 days of age on clinical and echocardiographic parameters

**DOI:** 10.1038/s41598-022-23418-z

**Published:** 2022-11-17

**Authors:** Amanda Sarita Cruz Aleixo, Danilo Otávio Laurenti Ferreira, Miriam Harumi Tsunemi, Simone Biagio Chiacchio, Maria Lucia Gomes Lourenço

**Affiliations:** 1 School of Veterinary Medicine and Animal Science, São Paulo State University (Unesp), Botucatu, São Paulo Brazil; 2 Secretary of Agriculture and Supply of the State of São Paulo - SAA/SP, Coordination of Agricultural Defense - CDA, Office of Agricultural Defense of Bauru - EDA, Bauru, Brazil; 3 Institute of Biosciences, São Paulo State University (Unesp), Botucatu, São Paulo Brazil; 4 School of Veterinary Medicine and Animal Science, São Paulo State University (Unesp), Rua Prof. Doutor Walter Mauricio Correa, S/N Bairro: Unesp Campus de Botucatu, Botucatu, São Paulo 18618-681 Brazil

**Keywords:** Physiology, Cardiology

## Abstract

The expansion of the sheep model in research represents an attractive and economically beneficial academic reason for investigations in sheep echocardiography. The present study aimed to evaluate the clinical and echocardiographic parameters in Dorper lambs during the developmental period. Emphasis was placed on the use of the species in translational research for the echocardiographic diagnosis of congenital heart diseases, which can contribute to improvements in interventionist techniques. Ten Dorper lambs were evaluated at the following time points: 24 h after birth and 7, 14, 21, 30, 60, 90 and 120 days of age. Clinical parameters were compiled, and echocardiogram records were obtained without sedation. Rectal temperature was lower on the first day compared to the others. From 21 days of life, there was a reduction in HR, with differences between time points. Mean and systolic blood pressure differed, with the highest values at 90 and 120 days of age. The thickness of the interventricular septum in diastole (IVSd) increased as age progressed, with the highest value at 120 days of age, and the same occurred for LVIDd (left ventricle internal diameter in diastole), LVFWd (left ventricular free wall thickness in diastole), IVSs (interventricular septum thickness in systole), LVIDs (left ventricle internal diameter in systole) and LVFWs (left ventricular free wall thickness in systole). There were differences in the size of the LA, Ao and LA/Ao ratio, which were greater at 90 days and 120 days of age. Echocardiographic changes accompany the development of lambs, where changes in echocardiographic parameters are evident with advancing age. The echocardiographic measurements in lambs obtained in the present study are similar to those in newborns.

## Introduction

Echocardiography is a noninvasive method for assessing the hearts of sheep and goats^1^. However, it is a technique that has been used more frequently in the evaluation of clinical conditions in small animals and in horses^2–4^ to assess changes in wall thickness, chamber size, appearance and valve function^5,6^. Studies have addressed cardiac dimensions in fetuses and lambs and cardiac function when valve implants are performed experimentally in the species^7–9^. Goats and sheep have been used as a model for heart failure (HF)^10,11^, and doxorubicin-induced HF is considered a reproducible and stable model species, a fact that is relevant for the study of chronic HF^12,13^.

Echocardiographic assessments in large animals were introduced by Pipes in the late 1970s, and echocardiographic images reproducible in horses^14^, cattle^15,16^ and pigs^17^ have been documented. Similar to swine, a recognized model for countless research efforts, sheep species have been increasingly used in cardiovascular research to evaluate cardiovascular parameters, including conduction pathways, fetal circulation, chronic heart disease and valve apparatuses^18^. This expansion of the sheep model represents an attractive and economically beneficial academic reason for investigations in sheep echocardiography^19^. However, the echocardiographic dimensions in lambs during development have not yet been well elucidated.

The development of animal models of cardiovascular diseases has provided new discoveries about the pathophysiological mechanisms of these diseases, contributing to the development of new therapies. Sheep are considered suitable for translational research, as the sheep heart is similar to the human heart and its dimensional and functional parameters are within human reference values. The objectives of the present study were to evaluate the clinical and echocardiographic parameters and their modifications in Dorper lambs 24 h after birth until 120 days of age, which may allow early identification of congenital heart disease by echocardiographic examination and contribute to interventional techniques.

## Materials and methods

### Study location

The present study was conducted at the Vale Verde site, Estrela do Vale hut, located in the city of Botucatu-SP, district of Rubião Júnior, latitude S-22.902107 and longitude W-48.516534, from December 2017 to April 2018. It was carried out according to animal welfare standards approved by the Ethics Committee on the Use of Animals of the Faculty of Veterinary Medicine and Zootechnics of the Universidade Estadual Paulista “Júlio de Mesquita Filho”, Botucatu Campus, under protocol CEUA-0174/2016 and was authorized by the owner who consented to the experimental plan and procedures performed.

### Animals

Ten Dorper lambs (*Ovis aries*) were evaluated from 24 h after birth to 120 days of age. For the evaluation of the lambs, they were separated from the mothers for data collection, and no anesthetic protocol was performed. Restraint was performed manually.

The clinical parameters compiled were HR (heart rate, beats per minute—0 bpm), respiratory frequency (RF) (movements per minute—mpm), rectal temperature (°C), capillary filling time (CFT), mucosal staining, systolic blood pressure (SBP), mean (MAP) and diastolic blood pressure (DBP) by the oscillometric method PetMap® (Blood Pressure Measurement Device. Ramsey Medical, Inc. Patent No. D531, 313 S), validated in the species according to Ulian et al. 2016^20^.

Assessments were performed in the morning, except on the first day after birth, which were performed 24 h after birth. The significance level was established at 0.05.

### Blood pressure measurement

The pressure measurement was performed on the left thoracic limb, not exceeding the dotted lines indicated on the cuff itself, and for the accuracy of the reading, the appropriate choice of the cuff was made according to the size of the animal's limb (40% of the limb diameter). The method detects the arterial pulse by means of oscillations and records HR values.

### Echocardiogram

The echocardiographic examination was obtained with the use of an ultrasound device (M-turbo Sonosite model) with a Doppler function and 2–8 MHz multifrequency sectorial transducer in 2D mode. In diastole, through the right parasternal window, the thickness of the interventricular septum (IVSd), left ventricle internal diameter (LVIDd) and thickness of the left ventricular free wall (LVFWd) were analyzed. In systole, interventricular septum thickness (IVSs), left ventricle internal diameter (LVIDs) and left ventricular free wall thickness (LVFWs) were analyzed. The left ventricular fraction shortening (LVFS), ejection fraction (EF), left atrial diameter (LA) in systole and diastolic aorta (Ao), and left atrium/aorta ratio (LA/Ao) were also compiled, along with pulmonary flow velocity (pul. velocity) and pulmonary pressure gradient (pres. grd.). The following formula was used to calculate LVFS (%): (LVIDd—LVIDs/LVIDd) × 100. It was not possible to attach the electrocardiograph´s cable to perform the echocardiogram, so measurements were taken in systole with the ventricle at its smallest diameter and in diastole with its largest diameter. The left atrium was measured at its greatest diameter at the end of systole. The means were calculated based on three measurements, all taken by the same operator (ASCA).

### Statistical analysis

The results are shown with the mean, standard deviation, minimum and maximum values obtained. For the analysis of the parameters, the normality test employed was Kolmogorov‒Smirnov; for the analysis of serial recordings, the Friedmann test was used. Friedmann's test was also used to assess possible significant differences between specific periods chosen from 24 h after birth to 21 days of age.

### Ethical approval

This study was conducted according to the animal well-being guidelines and approved by the Ethics Commission on Animal Use (CEUA, Comissão de Ética no Uso de Animais) of the School of Veterinary Medicine and Animal Science at Universidade Estadual Paulista “Júlio de Mesquita Filho”, Botucatu Campus, under protocol CEUA-0174/2016. The owners of the animals consented to the experimental plan and to the procedures performed. The project is in accordance with the precepts of Law No. 11.794, October 8, 2008, Decree No. 6.899, July 15, 2009, and the rules issued by the National Council for the Control of Animal Experimentation - CONCEA. The sheep used in this experiment belong to Sítio Vale Verde, Cabanha Estrela do Vale.

## Results

### Clinical parameters

**Figure 1:**
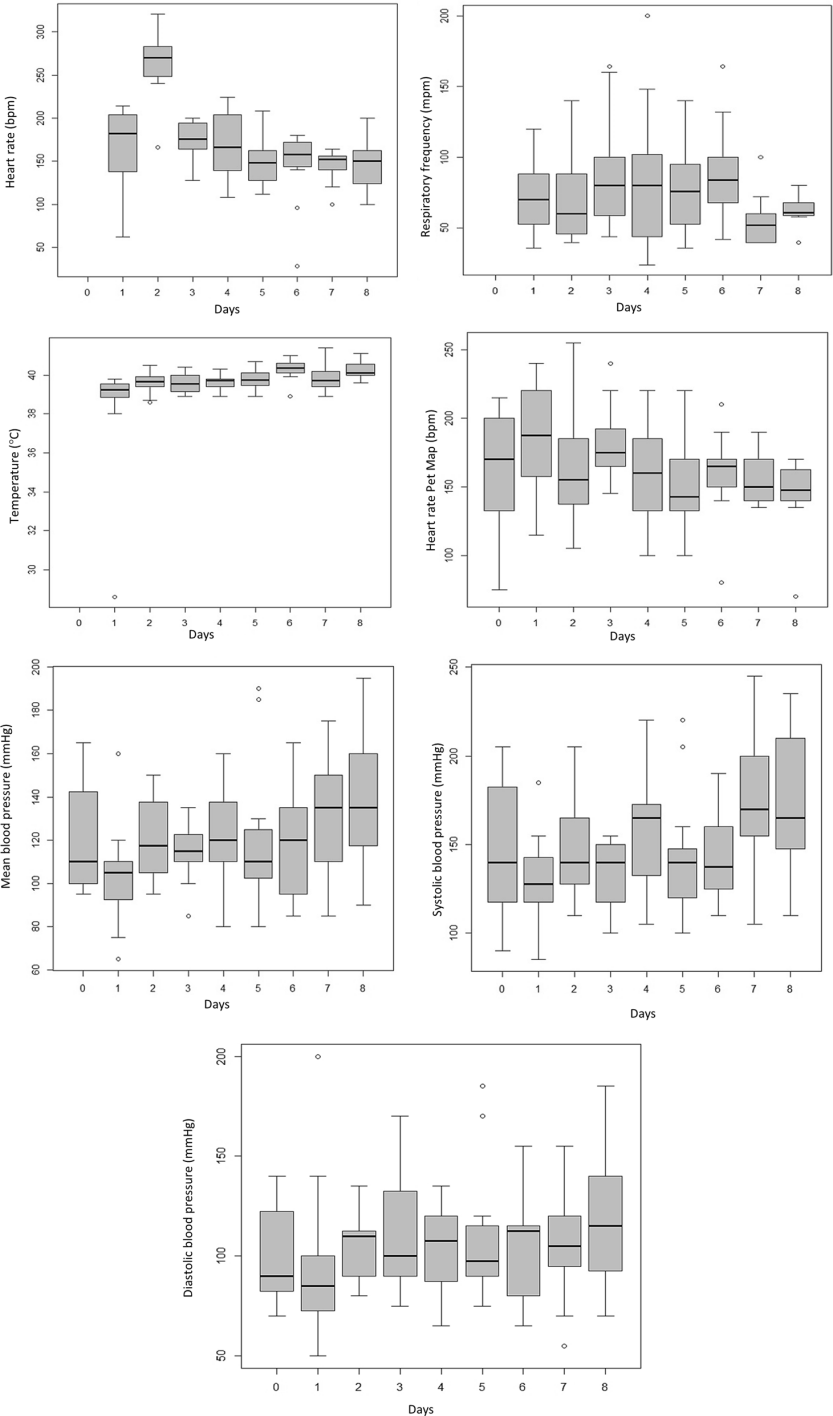
Clinical examination parameters during the neonatal period up to 120 days of age in Dorper lambs.

Table 1 and Fig. 1 illustrate the clinical examination parameters in lambs from 24 h after birth until 120 days of age. For the HR variable obtained by clinical examination, there was a significant difference between time points when HR was reduced from 21 days of age (HR—1 day: 173.00 ± 44.10, 7 days: 261.00 ± 37.34, 14 days: 170.00 ± 25.70, 21 days: 161.00 ± 36.35—*P* = 0.0001). There was no difference regarding RF. The rectal temperature differed significantly between time points (1 day: 38.30 ± 3.12, 7 days: 39.7 ± 0.50, 14 days: 39.60 ± 0.51, 21 days: 39.60 ± 0.30—P = 0.0001), and after 7 days, there was a gradual increase, with the highest values observed at 60 (40.20 ± 0.53 °C) and 120 days of age (40.20 ± 0.40 °C).

**Table 1:** Clinical examination parameters (mean, standard deviation, minimum, and maximum) in lambs from 24 h after birth until 120 days of age.

Clinical Parameters	2 h after birth	1 day	7 days	14 days	21 days	30 days	60 days	90 days	120 days	*P*
HR (bpm)	–	173.00 ± 44.10^a^ (62;214)	261.00 ± 37.34^b^ (166;321)	170.00 ± 25.707^ac^ (128;200)	161.00 ± 36.35^ac^ (108;220)	146.00 ± 25.78 (112;208)	143.00 ± 42.53 (128;180)	142.00 ± 18.49 (100;164)	146.00 ± 26.93 (100;200)	*0.000
RF (mpm)	–	71.00 ± 23.46 (36;120)	70.00 ± 32.28 (40;140)	85.00 ± 35.71 (44;164)	82.00 ± 49.00 (24;200)	76.00 ± 29.08 (36;140)	89.00 ± 31.83 (42;164)	57.00 ± 22.33 (40;100)	60.00 ± 12.40 (40;80)	0.119
T (°C)	–	38.30 ± 3.12^a^ (38.6;39.8)	39.7 ± 0.50^b^ (38.6;40.5)	39.60 ± 0.51^b^ (38.9;40.4)	39.60 ± 0,30^b^ (38.9;40.3)	39.80 ± 0.42 (39.1;40.7)	40.20 ± 0.53 (38;9.41)	39.80 ± 0.59 (39.3;41.4)	40.20 ± 0.40 (39.6;41.1)	*0.000
MUCOSAL	–	1	1	1	1	1	1	1	1	–
CFT SBP	–	2	2	2	2	2	2	2	2	–
Diastolic	103.75 ± 27.15 (70;140)	87.50 ± 23.11 (50;140)	103.75 ± 19.67 (80;135)	112.50 ± 35.19 (75;170)	99.58 ± 20.05 (65;130)	110.83 ± 34.23 (75;185)	102.92 ± 27.84 (65;155)	103.75 ± 28.13 (55;155)	117.92 ± 35.19 (70;185)	0.193
Mean	126.67 ± 25.79 (95;165)	102.92 ± 23.60^a^ (65;160)	120.83 ± 19.10^b^ (95;150)	113.33 ± 12.85 ^b^ (85;135)	118.75 ± 22.97^b^ (80;160)	123.75 ± 33.04 (80;190)	116.67 ± 25.88 (85;165)	131.67 ± 28.31 (85;175)	138.75 ± 32.48 (90;195)	*0.026
Systolic	162.08 ± 31.15 (115;205)	130.42 ± 26.06 ^a^ (85;185)	146.67 ± 28.07 ^b^ (110;205)	130.83 ± 20.09^ab^ (100;155)	154.58 ± 32.08^c^ (105;220)	145.83 ± 35.47 (100;220)	141.67 ± 25.26 (110;190)	174.17 ± 38.78 (105;245)	173.75 ± 39.61 (110;235)	*0.002
HR Pet Map	160.00 ± 46.63 (75;215)	180.00 ± 44.44^a^ (115;240)	168.00 ± 44.22^b^ (105;255)	182.00 ± 29.58^c^ (145;240)	155.00 ± 36.31^b^ (100;220)	143.33 ± 28.95 (100;220)	157.00 ± 28.72 (80;190)	152.00 ± 15.30 (135;175)	145.00 ± 26.63 (70;170)	*0.031

Kolmogorov‒Smirnov normality test; comparison between Friedmann's test moments; * significance: *p* < 0.05; HR- heart rate; RF- respiratory frequency; T- rectal temperature; CFT- capillary filling time; SBP- systolic blood pressure; ^ab^ letters overwritten on the same line indicate a significant difference between specific moments (24 h after birth up to 21 days of age).

Mean blood pressure differed between time points (1 day: 126.67 ± 25.79, 7 days: 120.83 ± 19.10, 14 days: 113.33 ± 12.85, 21 days: 118.75 ± 22.97—*P* = 0.026), with the highest values at 90 (131.67 ± 28.31 mmHg) and 120 days (138.75 ± 32.48 mmHg) of age, and for systolic blood pressure, there was a significant difference between 24 h after birth and at 7 and 21 days and a significant difference between 7 days at 21 days and 14 days at 21 days of age (1 day: 162.08 ± 31.15, 7 days: 130.42 ± 26.06, 14 days: 130.83 ± 20.09, 21 days: 154.58 ± 32.08—*P* = 0.002). FC Petmap® did not differ significantly between 7 and 21 days, with a difference between 1 and 7 days; 14 days differed between 7 and 21 days (1 day: 180.00 ± 44.44, 7 days: 168.00 ± 44.22, 14 days: 182.00 ± 29.58, 21 days: 155.00 ± 36.31—*P* = 0.031).

### Echocardiographic parameters

**Figure 2:**
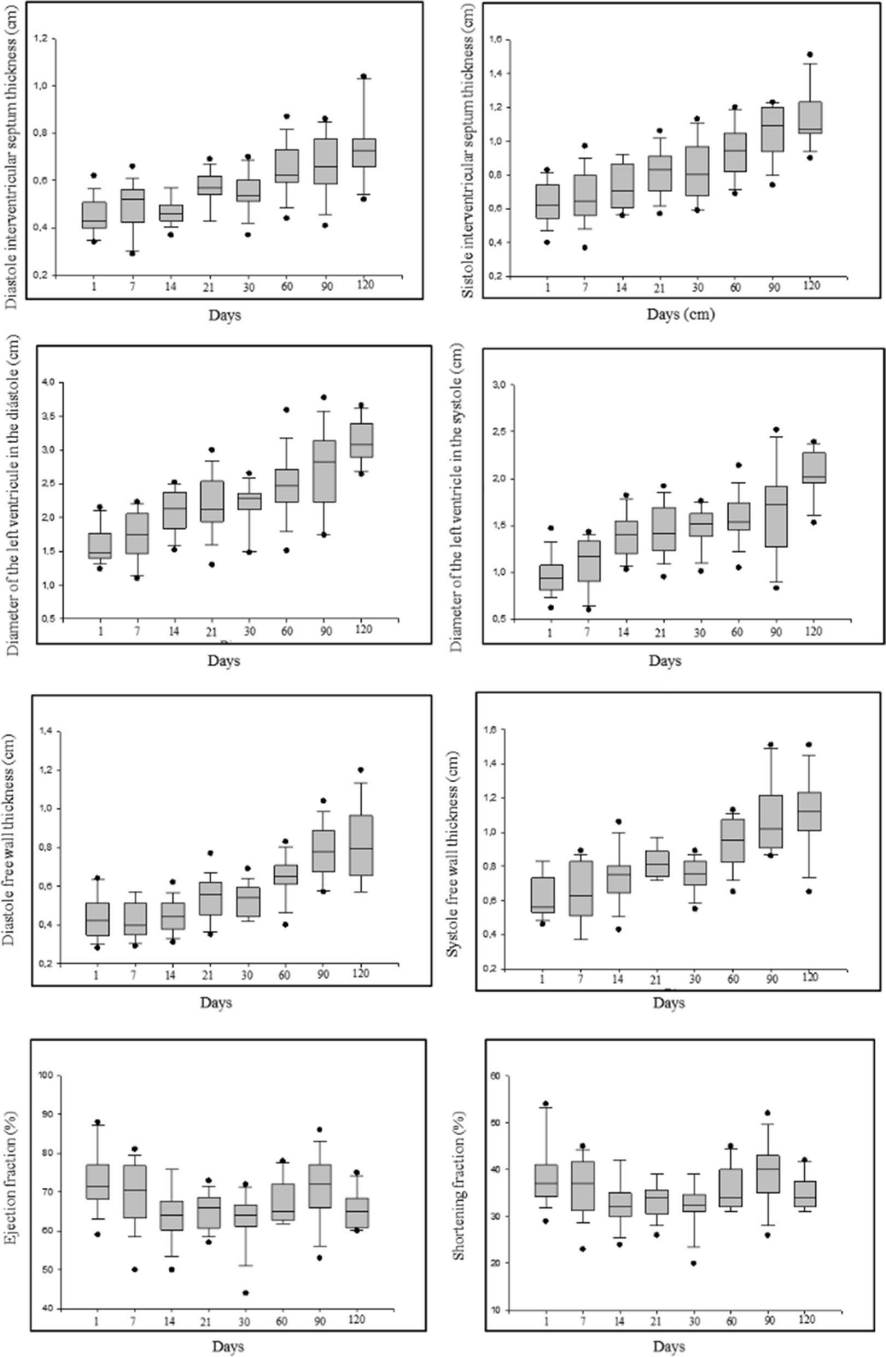

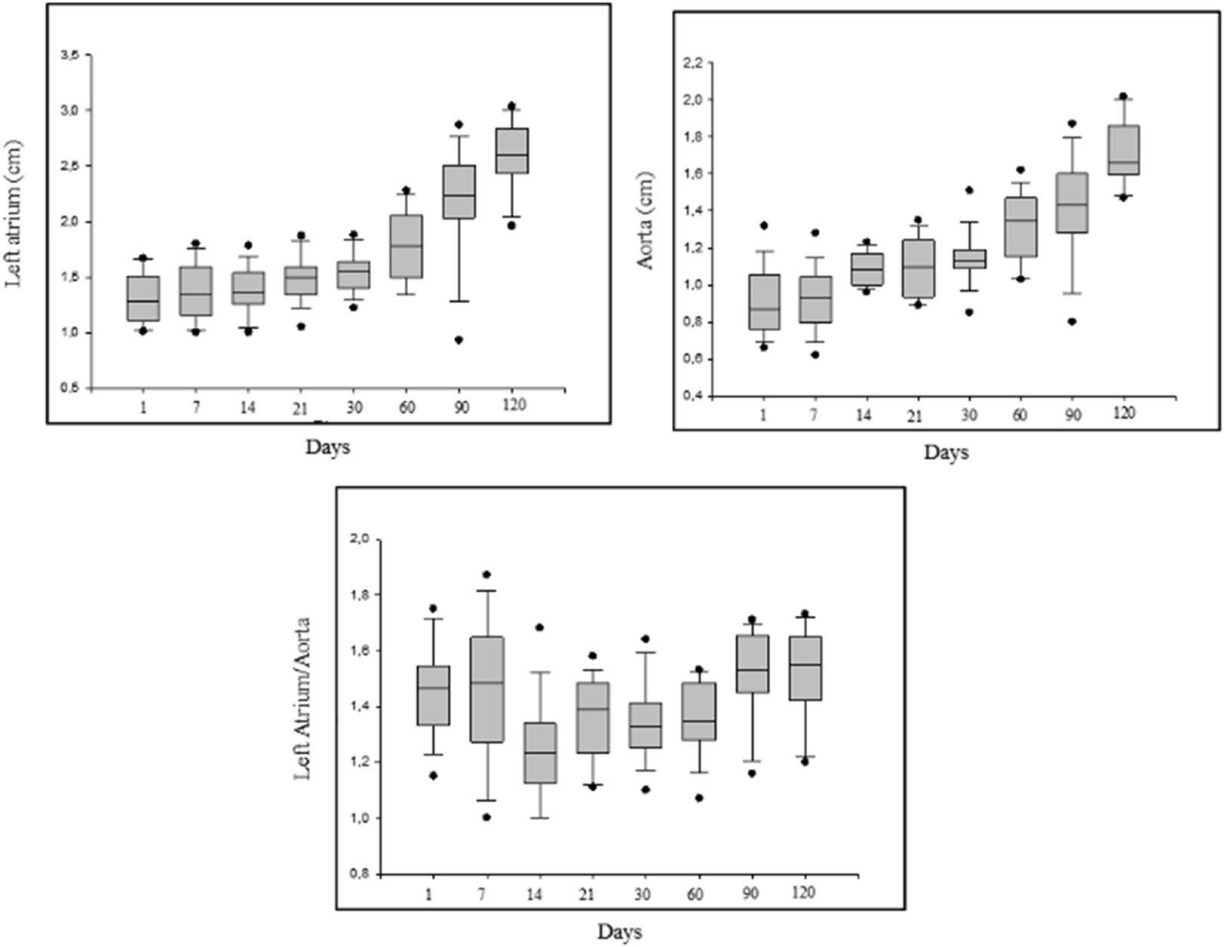
Echocardiographic parameters during the neonatal period up to 120 days of age in Dorper lambs.

Table 2 and Fig. 2 illustrate the echocardiographic parameters in lambs from 24 h of birth to 120 days of age. The thickness of the IVSd gradually increased as age progressed (1 day: 0.46 ± 0.08, 7 days: 0.48 ± 0.10, 14 days: 0.46 ± 0.05, 21 days: 0.58 ± 0.07—*P* = 0.0001), with the highest value at 120 days of age (0.75 ± 0.15), and the same occurred for LVIDd (1 day; 1.62 ± 0.27, 7 days: 1.77 ± 0.33, 14 days: 2.15 ± 0.28, 21 days: 2.29 ± 0.32—*P* = 0.0001), LVFWd (1 day: 0.45 ± 0.12, 7 days: 0.43 ± 0.09, 14 days: 0.45 ± 0.09, 21 days: 0.56 ± 010—*P* = 0.0001), IVSs (1 day: 0.64 ± 0.12, 7 days: 0.66 ± 015, 14 days: 0.73 ± 0.13, 21 days: 0.84 ± 0.10—*P* = 0.0001), LVIDs (1 day: 0.96 ± 0.20, 7 days: 1.10 ± 0.26, 14 days: 1.39 ± 0.26, 21 days: 1.45 ± 0.27—*P* = 0.0001) and LVFWs (1 day: 0.63 ± 0.13, 7 days: 0.67 ± 0.14, 14 days: 0.76 ± 0.14, 21 days: 0.84 ± 0.09—*P* = 0.0001), with a significant difference for the referred parameters in different age groups.

**Table 2:** Echocardiographic parameters (mean, standard deviation, minimum, and maximum) in lambs from 24 h of birth to 120 days of age.

Echocardiographic Parameters	24 h	7 days	14 days	21 days	30 days	60 days	90 days	120 days	*P*
IVSd (cm)	0.46 ± 0.08^a^ (0.34; 0.62)	0.48 ± 0.10^b^ (0.29; 0.59)	0.46 ± 0,05^c^ (0.37; 0.56)	0.58 ± 0.07^b^ (0.43; 0.69)	0.56 ± 0.07 (0.44;0.70)	0.64 ± 0.11 (0.44;0.87)	0.67 ± 0.13 (0.41;0.86)	0.75 ± 0.15 (0.52;1.04)	*0.000
LVIDd (cm)	1.62 ± 0.27^a^ (1.35;2.15)	1.77 ± 0.33^b^ (1.10;2.23)	2.15 ± 0.28^c^ (1.60;2.52)	2.29 ± 0.32^c^ (1.89;3.00)	2.33 ± 0.18 (2.04;2.65)	2.56 ± 0.41 (2.08;3.59)	2.76 ± 0.56 (1.74;3.77)	3.14 ± 0.32 (2.64;3.66)	*0.000
LVFWd (cm)	0.45 ± 0.12^a^ (0.28;0.64)	0.43 ± 0.09^a^ (0.31;0.57)	0.45 ± 0.09^a^ (0.31;0.62)	0.56 ± 0.10^b^ (0.37;0.77)	0.53 ± 0.08 (0.42;0.69)	0.65 ± 0.11 (0.40;0.83)	0.80 ± 0.13 (0.59;1.04)	0.82 ± 0.19 (0.57;1.20)	*0.000
IVSs (cm)	0.64 ± 0.12^a^ (0.40;0.83)	0.66 ± 0.15^a^ (0.37;0.87)	0.73 ± 0.13^b^ (0,56;0,92)	0.84 ± 0.10^c^ (0.69;1.06)	0.85 ± 0.17 (0.60;1.13)	0.95 ± 0.16 (0,69;1,20)	1.07 ± 0.15 (0,74;1,23)	1.13 ± 0.16 (0,90;1,51)	*0.000
LVIDs (cm)	0.96 ± 0.20^a^ (0.62;1.47)	1.10 ± 0.26^b^ (0.60;1.43)	1.39 ± 0.26^c^ (1.03;1.82)	1.45 ± 0.27^c^ (0.95;1.92)	1.48 ± 0.20 (1.01;1.76)	1.57 ± 0.24 (1.05;2.14)	1.65 ± 0.49 (0.83;2.52)	2.04 ± 0.24 (1.53;2.39)	*0.000
LVFWs (cm)	0.63 ± 0.13^a^ (0.46;0.83)	0.67 ± 0.14^b^ (0.49;0.89)	0.76 ± 0.14^c^ (0.54;1.06)	0.84 ± 0.09^d^ (0.72;0.97)	0.77 ± 0.08 (0.63;0.89)	0.95 ± 0.15 (0.65;1.13)	1.09 ± 0.22 (0.86;1.51)	1.11 ± 0.21 (0.65;1.51)	*0.000
EF (%)	72.00 ± 7.42^a^ (59.00;88.00)	69.00 ± 8.18^b^ (50.00;81.00)	64.00 ± 6.99^c^ (50.00;76.00)	65.00 ± 4.67^c^ (57.00;73.00)	63.00 ± 6.60 (44.00;72.00)	67.00 ± 5.67 (62.00;78.00)	71.00 ± 8.56 (53.00;86.00)	65.00 ± 4.74 (60.00;75.00)	*0.010
LVFS (%)	38.00 ± 6.68^a^ (29.00;54.00)	36.56 ± 5.96^b^ (23.00;45.00)	33.00 ± 5.25^c^ (24.00;42.00)	33.00 ± 3.63^c^ (26.00;39.00)	32.00 ± 4.72 (20.00;39.00)	35.00 ± 4.79 (31.00;45.00)	39.00 ± 6.60 (26.00;52.00)	34.00 ± 3.76 (31.00;42.00)	*0.029
LA (cm)	1.33 ± 0.20^a^ (1.03;1.66)	1.38 ± 0.25^a^ (1.00;1.73)	1.43 ± 0.22^a^ (1,06;1,78)	1.51 ± 0.16^b^ (1.29;1.81)	1.55 ± 0.15 (1.32;1.82)	1.85 ± 0.29 (1.34;2.28)	2.30 ± 0.29 (1.82;2.87)	2.59 ± 0.30 (1.96;3.04)	*0.000
Ao (cm)	0.91 ± 0.14^a^ (0.70;1.12)	0.96 ± 0.14^a^ (0.76;1.28)	1.11 ± 0.09^b^ (0.96;1.23)	1.13 ± 0.14^b^ (0.91;1.35)	1.18 ± 0.12 (1.02;1.51)	1.33 ± 0.18 (1.03;1.62)	1.48 ± 0.19 (1.18;1.87)	1.70 ± 0.17 (1.47;2.02)	*0.000
LA/Ao	1.47 ± 0.18^a^ (1.15;1.75)	1.40 ± 0.24^a^ (1.00;1.79)	1.28 ± 0.18^b^ (1.06;1.68)	1.35 ± 0.13^c^ (1.11;1.51)	1.32 ± 0.12 (1.10;1.57)	1.39 ± 0.10 (1.25;1.53)	1.55 ± 0.13 (1.27;1.71)	1.52 ± 0.16 (1.20;1.73)	*0.000
Aortic flow Velocity (cm/s)	91.57 ± 13.73 (68.40;111.8)	80.41 ± 11.26 (66.80;98.5)	86.71 ± 17.59 (65.90;108.50)	82.46 ± 8.84 (69.30;92.70)	77.38 ± 7.50 (68.40;90.10)	82.23 ± 12.01 (66.80;106.00)	76.32 ± 4.54 (71.00;82.60)	84.49 ± 12.22 (67.50;105.50)	0.119
Pres. grd.	3.42 ± 1.00 (1.87;5.00)	2.63 ± 0.73 (1.78;3.88)	3.09 ± 1.20 (1.74;4.64)	2.64 ± 0.52 (1.92;3.44)	2.42 ± 0.47 (1.87;3.25)	2.74 ± 0.80 (1.78;4.36)	2.34 ± 0.28 (2.01;2.73)	2.91 ± 0.85 (1.82;4.45)	0.098
Pulmonary flow Velocity (cm/s)	86.33 ± 19.59 (62.60;123.50)	77.48 ± 15.25 (60.10;104.00)	79.58 ± 14.10 (60.10;103.50)	78.60 ± 13.81 (64.30;106.00)	71.83 ± 5.95 (65.10;83.90)	81.64 ± 12.96 (61.00;101.20)	78.96 ± 14.08 (60.30;106.00)	76.67 ± 8.70 (63.90;94.00)	0.270
Pres. grd.	3.12 ± 1.45 (1.57;6.10)	2.49 ± 1.00 (1.44;4.35)	2.61 ± 0.94 (1.44;4.28)	2.54 ± 0.95 (1.65;4.49)	2.08 ± 0.35 (1.70;2.82)	2.70 ± 0.81 (1.49;4.10)	2.57 ± 0.92 (1.45;4.51)	2.38 ± 0.54 (1.63;3.53)	0.270

Kolmogorov‒Smirnov normality test; comparison between Friedmann's test moments; * significance: *p* < 0.05; IVS- interventricular septum; LVID- left ventricle internal diameter; LVFW: left ventricle free wall; d—diastole; s—systole; EF: ejection fraction; LVFS: left ventricular fraction shortening; LA: left atrium; Ao: aorta; Pres. grd.: pressure gradient; ^ab^ different letters superscript on the same line indicate a significant difference between specific moments (24 h after birth up to 21 days of age).

**Figure 3:**
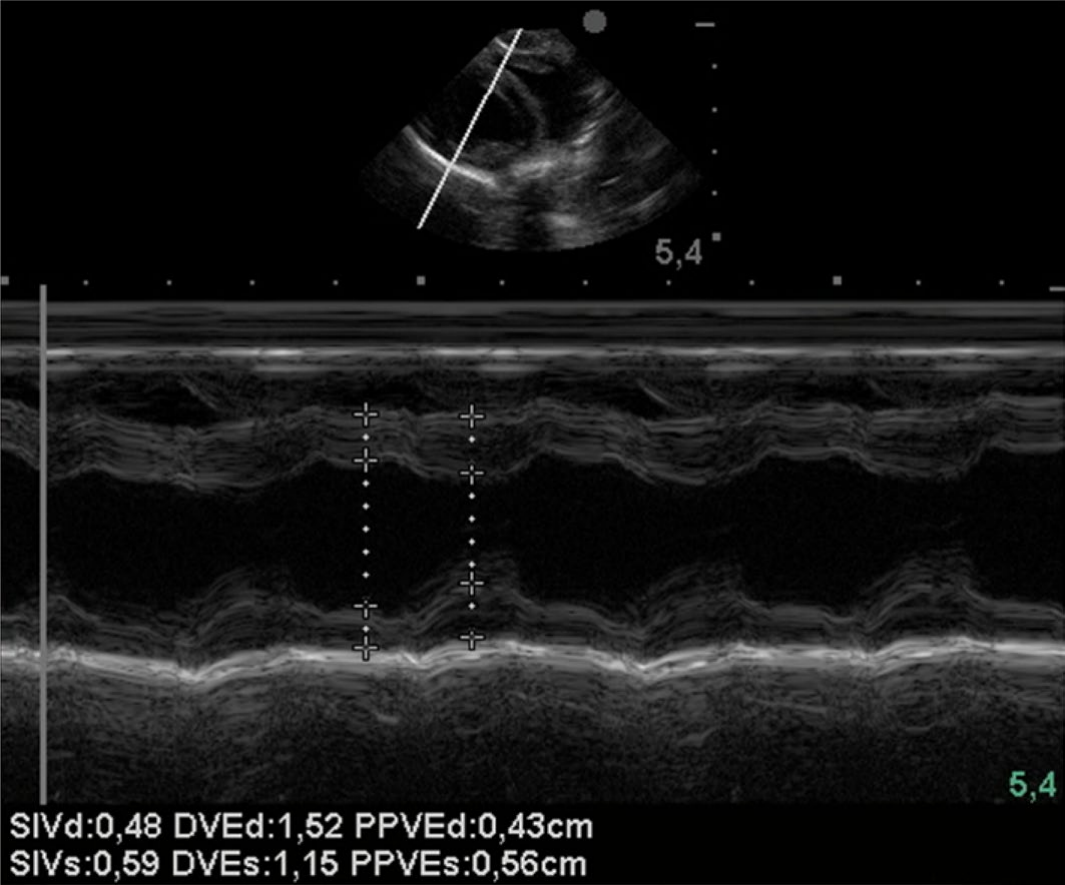
Echocardiographic evaluation, M-mode, left ventricular diameter end-diastolic and end-systolic.

**Figure 4:**
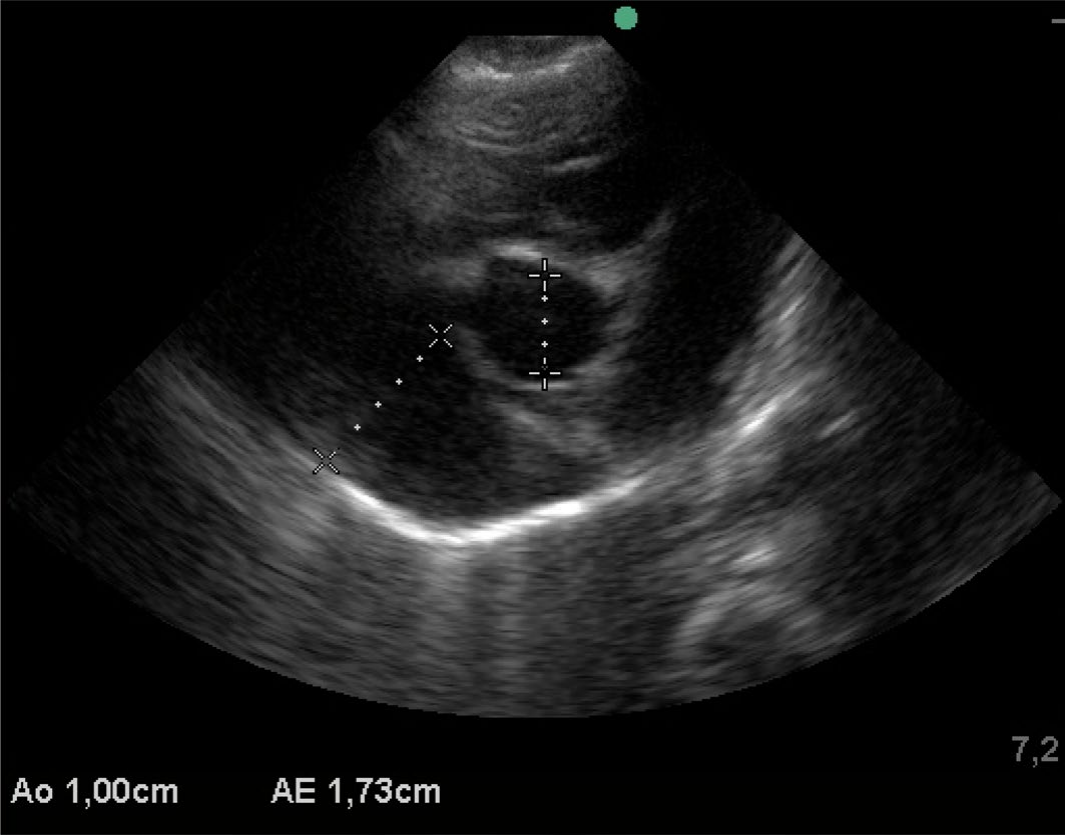
Echocardiographic evaluation, two-dimensional mode, and left atrium/aorta ratio.

There was a significant difference for the size of the LA, Ao and LA/Ao ratio, which was greater at 90 (LA: 2.30 ± 0.29, LA/Ao: 1.55 ± 0.13) days and 120 (LA: 2.59 ± 0.30, LA/Ao: 1.52 ± 0.16) days of age. Figure 3 shows echocardiographic evaluation, M-mode, left ventricular diameter end-diastolic and end-systolic. EF and LVFS differed during the age periods, with the lowest values at 14 and 30 days of age for both parameters. The size of the LA and diameter of the aorta differed significantly at different time points analysed, with the LA dimension gradually increasing with age. There was also a significant difference in the LA/Ao ratio (1 day: 1.47 ± 0.18, 7 days: 1.40 ± 0.24, 14 days: 1.28 ± 0.18, 21 days: 1.35 ± 0.13—*P* = 0.0001). There was no difference in the time points analyzed regarding flow and pulmonary velocity and their respective pressure gradients. Figure 4 shows the echocardiographic evaluation, two-dimensional mode, left atrium/aorta ratio.

In the evaluation of echocardiographic parameters at the specific time points chosen 24 h after birth until 21 days of age, there were significant differences in diastole: IVS differed between all the mentioned time points, except between 7 and 21 days, which did not differ. There was a difference in LVIDd between all those time points, except between 14 and 21 days; for LVFW, there was a difference between 24 h after birth and 21 days, 7 days and 21 days, and 14 days and 21 days of age. In systole, the IVS differed between all the referred time points except between 24 h after birth and 7 days of age, LVIDs also differed between all the referred time points except between 14 and 21 days, and LVFW differed between all the different time points.

EF (1 day: 72.00 ± 7.42, 7 days: 69.00 ± 8.18, 14 days: 64.00 ± 6.99, 21 days: 65.00 ± 4.67—*P* = 0.010) and LVFS (1 day: 38.00 ± 6.68, 7 days: 36.56 ± 5.96, 14 days: 33.00 ± 5.25, 21 days: 33.00 ± 3.63—*P* = 0.029) showed significant differences between all time points mentioned, except between 14 and 21 days. The size of the LA differed between 24 h after birth and 21 days, 7 days and 21 days, and 14 and 21 days. The diameter of the aorta differed between all referred time points (24, 7, 14 and 21 days), except between 24 h and 7 days and between 14 and 21 days. The LA/Ao ratio differed between all of the aforementioned time points except between 24 h after birth and 7 days of age.

## Discussion

This study has demonstrated for the first time longitudinal cardiovascular parameters, including cardiac structural and functional parameters, in Dorper lambs.

### Clinical parameters

#### HR and blood pressure

We observed changes in HR as age progressed, probably as a result of increased vagal activity as development proceeds. After 21 days, there was a reduction in HR in lambs. Siimes et al. 1990^21^ reported that in newborns, both divisions of the SNA regulate HRV, and subsequently, there is a predominance of vagal activity because of the maturation of the SNA, where reductions in HR in lambs during development reflect the development of the ANS. Since blood pressure values are directly related to HR, despite the reduction in HR at 21 days, the same did not occur with blood pressure values, where we noticed increases in pressures as development occurred. This fact may be related to HR variations during the day and to the handling of animals.

We observed higher blood pressure values in lambs 2 h after birth and in the first 24 h after birth. The ovine fetus has been useful as a model for studying the maturation of fetal organs, as it has similarities in the maturational trajectory with humans^22^, which is partly due to the similar peak in cortisol production in the days before birth. These findings reflect the cardiovascular adaptations associated with birth and the transition to extrauterine life^23^.

The output doubles approximately after birth as the ventricular circulation is modified from a parallel (fetal) circuit to a series circuit (infant/adult). Systolic blood pressure also increases at birth^24^, partly because of the removal of the low-resistance placental unit, increased systemic vascular resistance and circulating vasoactive hormones, including cortisol and catecholamines. These adaptations are believed to provide an increase in metabolic demand and processes that regulate breathing and thermogenesis, which is reflected by changes in blood flow to certain organs during this transition^25^.

The rectal temperature was elevated after 60 days of age in lambs, and the highest values of MAP and SBP were observed after 21 days of age. The variations in pressure values found in our study may be due to variations in HR throughout the day. Kovács et al. 2016^26^, in their study aiming to analyze circadian rhythm in cattle, observed an evolution throughout the day of HR in the summer, starting with an extreme increase from 7:00 to 17:00 h and decreasing from 18:00.

The authors reported that SNA activity showed a circadian rhythm in summer but not in winter, where they observed an increase in sympathetic tone during the day and a considerable increase in parasympathetic tone during the night in summer. The authors believe that the reduction in sympathetic activity from the end of the afternoon with a parallel increase in parasympathetic tone would be associated with a reduction in ambient temperature in the summer. Thus, the high values of HR, blood pressure and temperature can be secondary to greater sympathetic activation during the day, where our collections were performed only during that period.

### Echocardiographic parameters

#### LVID, IVS, LVFW

The echocardiographic parameters showed changes as the lambs age, where there was a progressive increase in them. A similar phenomenon occurs in the human species, demonstrating that development advances, and consequently, the body surface area increases. There are changes in echocardiographic parameters, and this factor needs to be considered for the analysis in lambs.

As in our study, the studies by Poser et al. 2013^27^ and Lago et al. 2009^28^ in lambs during growth showed that age group was a significant factor for echocardiographic parameters and that changes in echocardiography can occur up to one year of life. LVIDd and LVIDs progressively increased with age, and the same occurred for LVFW and IVS. The progressive changes in echocardiographic parameters in our study may also be due to the development of cardiomyocytes. After birth, the heart undergoes intense growth and remodeling driven by cardiomyocyte hyperplasia^29^, increased density and organization of myofibrils, maturation of the mechanical and electrical coupling between cardiomyocytes and the appearance of binucleated cells^30^.

The influence of age itself should be considered to a lesser extent; however, as age advances, the body surface area increases, and the changes in the echocardiographic parameters observed in the present study are related to the body surface area, as mentioned by Knutsen et al. (1989)^31^, which proved that the body surface area should be considered for the interpretation of echocardiographic parameters in men and women.

#### Doppler study

In our study, no significant effect of development was found for parameters derived from Doppler.

#### EF and LVFS

The EF obtained by echocardiogram was high, where such findings may be due to hemodynamic changes because of the transition from fetal to neonatal life. During the first months of life, there is a reduction in the concentration of hemoglobin, called physiological anemia, as it occurs in the absence of any recognized nutritional deficiencies. This reduction is offset by the greater extraction of O_2_ from hemoglobin. Simultaneously, with these hematological changes, there are high metabolic demands imposed by the extrauterine environment. Therefore, for tissue oxygenation to remain adequate, there must be an improved O2 extraction and an increase in cardiac output^32^. We believe that there was no difference in flow assessments in our study due to physiological adaptations that occur proportionally to development, but studies are needed to evaluate and compare hematological and echocardiographic parameters in lambs during development.

LVFS and EF were higher at 24 h after birth than at all evaluated time points. In the periods analyzed during lamb development by Poser et al. 2013^27^, there was variation in the behavior of the systolic function indices (LVFS and EF), but there was no significant difference. In our study, there were differences between the time points evaluated, a fact that did not occur in the study of the referred authors, who evaluated the animals under the effect of sedation. We believe that the effect of the anesthetic protocol contributes to this result; as development progresses in lambs, the systolic function indices stabilize after 60 days of age. In addition, the effect of anesthetic protocols should also be considered in addition to age for the analysis of echocardiographic parameters in lambs.

The ovine species has been widely used in cardiovascular research because of its characteristics, which are similar to those of the human heart^33^. The clinical evaluation of lambs during development and the use of echocardiography reflect the innumerable changes that occur during this period and provide relevant information for the species, which can contribute to early illustration of physiological changes that may limit the development of the species and its use in the areas of production and reproduction.

The echocardiogram also proved to be effective in the present study to illustrate the hemodynamic changes that occur during development, which contribute to the early diagnosis of congenital heart disease, enabling the use of the species in research aimed at interventional techniques prior to the development of clinical signs.

#### Study limitations

This study has some limitations. The relatively small sample size of 10 animals may have influenced our results, especially with regard to nonsignificant data. Echocardiographic analyses were also performed using simple methods, but analyses employing more advanced modalities of echocardiography could contribute with greater magnitude to the assessment of the influence of development on echocardiographic parameters. Additionally, the assessment of body surface area was not considered in the present study for the evaluation of echocardiographic parameters, which could illustrate more clearly the influence of body size on the exam. It was not possible to attach the electrocardiogram cable to perform the echocardiogram, which is also a limitation of the study.

## Conclusions

During development, the cardiovascular system undergoes numerous changes, which can be reproduced by systemic blood pressure and visualized by echocardiography in lambs. The species is widely used in medical research since echocardiographic parameters change from birth and progress during growth and are similar to those in newborns. The sheep species proved to be useful to reproduce such parameters and to investigate heart diseases that may occur in this phase, allowing advances in diagnosis and intervention.

## Data Availability

The datasets are available from the corresponding author on reasonable request.

## Abbreviations

HR: Heart rate
HF: Heart failure
RF: Respiratory frequency
IVSd: Interventricular septum in diastole
LVIDd: Left ventricle internal diameter in diastole
LVFWd: Left ventricular free wall in diastole
IVSs: Interventricular septum systole
LVIDs: Left ventricle internal diameter in systole
LVFWs: Left ventricular free wall in systole
CFT: Capillary filling time
SBP: Systolic blood pressure
MAP: Mean arterial pressure
DBP: Diastolic blood pressure
LVFS: Left ventricular fraction shortening
EF: Ejection fraction
LA: Left atrium
Ao: Aorta
Pres. grd.: Pressure gradient
mpm: Movements per minute
bpm: Beats per minute
